# Detecting and localizing cervical lesions in colposcopic images with deep semantic feature mining

**DOI:** 10.3389/fonc.2024.1423782

**Published:** 2024-11-22

**Authors:** Li Wang, Ruiyun Chen, Jingjing Weng, Huiping Li, Shi Ying, Jinghui Zhang, Zehao Yu, Chengbin Peng, Siming Zheng

**Affiliations:** ^1^ Gynaecology Department, Ningbo Medical Centre Lihuili Hospital, Ningbo, Zhejiang, China; ^2^ College of Information Science and Engineering, Ningbo University, Ningbo, Zhejiang, China; ^3^ Ningbo Weijie Medical Technology Co., LTD, Ningbo, Zhejiang, China; ^4^ Health Science Center, Ningbo University, Ningbo, Zhejiang, China; ^5^ Hepatopancreatobiliary Surgery Department, The First Affiliated Hospital of Ningbo University, Ningbo, Zhejiang, China

**Keywords:** cervical lesions, colposcopy, artificial intelligence, deep learning, regional localization

## Abstract

**Objective:**

This study aims to investigate the feasibility of employing artificial intelligence models for the detection and localization of cervical lesions by leveraging deep semantic features extracted from colposcopic images.

**Methods:**

The study employed a segmentation-based deep learning architecture, utilizing a deep decoding network to integrate prior features and establish a semantic segmentation model capable of distinguishing normal and pathological changes. A two-stage decision model is proposed for deep semantic feature mining, which combines image segmentation and classification to categorize pathological changes present in the dataset. Furthermore, transfer learning was employed to create a feature extractor tailored to colposcopic imagery. Multi-scale data were bolstered by an attention mechanism to facilitate precise segmentation of lesion areas. The segmentation results were then coherently mapped back onto the original images, ensuring an integrated visualization of the findings.

**Results:**

Experimental findings demonstrated that compared to algorithms solely based on image segmentation or classification, the proposed approach exhibited superior accuracy in distinguishing between normal and lesioned colposcopic images. Furthermore, it successfully implemented a fully automated pixel-based cervical lesion segmentation model, accurately delineating regions of suspicious lesions. The model achieved high sensitivity (96.38%), specificity (95.84%), precision (97.56%), and f1 score (96.96%), respectively. Notably, it accurately estimated lesion areas, providing valuable guidance to assisting physicians in lesion classification and localization judgment.

**Conclusion:**

The proposed approach demonstrates promising capabilities in identifying normal and cervical lesions, particularly excelling in lesion area segmentation. Its accuracy in guiding biopsy site selection and subsequent localization treatment is satisfactory, offering valuable support to healthcare professionals in disease assessment and management.

## 1 Introduction

Cervical cancer stands as one of the most prevalent malignancies affecting women globally (1), while the persistent high-risk HPV infection (2) is the main cause of most cervical precancer and cervical cancers. The introduction of the “three-step cervical cancer screening” process in the 1950s—comprising cytology and/or human papillomavirus (HPV) testing as the first step, followed by colposcopy and then histopathology—has enabled the early detection and treatment of cervical cancer and its precancerous conditions (3–5). Colposcopy plays a pivotal role in the timely diagnosis of cervical cancer and precancerous lesions (6–9).

Presently, colposcopy relies on a comprehensive description system for colposcopic images (10–12), meticulously defining and categorizing cervical epithelium and blood vessels based on their borders, contours, morphology, and other tissue characteristics (13–15). However, the diagnostic accuracy of colposcopy varies widely due to differences in expertise among colposcopists, impacting the standardized treatment of cervical lesions and potentially resulting in over- or under-treatment (16–18).

In recent years, deep learning (DL) has emerged as a promising tool to enhance the diagnostic efficiency and standardization of medical images, leveraging its robust feature mining capabilities (19). Nonetheless, prior studies predominantly focus on classifying specific lesions across entire images, with limited research on localizing and identifying lesion regions (20–23). Consequently, practical clinical applications have seen minimal advancement. In clinical practice, while lesion characterization can be validated through final histopathology, colposcopic localization for guiding biopsy or treatment remains crucial, fulfilling indispensable clinical needs.

This study employs DL for colposcopic image segmentation and feature recognition to analyze image features across various lesion levels, thereby acquiring standardized information regarding the progression of specific lesions. Furthermore, it establishes a feature extractor through transfer learning from clinically significant colposcopic image data within a two-stage architecture, facilitating combined image segmentation. The findings not only hold relevance for clinical screening but also offer guidance for biopsy and subsequent localization treatment.

## 2 Materials and methods

### 2.1 Study subjects

A total of 1837 abnormal colposcopic images were collected from Ningbo Medical Center Lihuili Hospital and The First Affiliated Hospital of Ningbo University, comprising 1124 images depicting low-grade squamous intraepithelial lesions, 575 images depicting high-grade squamous intraepithelial lesions, and 138 images depicting cervical cancer. These images were annotated according to the standardized colposcopic terminology established by the International Federation for Cervical Pathology and Colposcopy and the American Society for Colposcopy and Cervical Pathology. Additionally, 1070 normal colposcopic images were included for accurate lesion detection.

### 2.2 Data collection and labeling

#### 2.2.1 Data collection

Colposcopic images, along with pertinent patient information including age, ThinPrep cytologic test (TCT) results, and human papillomavirus (HPV) status, were retrospectively gathered from January 2018 to December 2021 at the Ningbo Medical Center Lihuili Hospital. Colposcopy was conducted using a photoelectric all-in-one digital electronic colposcope (Feinmechanik-Optik GmbH) from LEISEGANG, Germany, equipped with a Canon EOS600D camera. To mitigate model bias, additional colposcopic images and patient data were obtained from The First Affiliated Hospital of Ningbo University, utilizing a high-definition colposcope (EDAN C6HD) from China.

Colposcopy procedures included a conventional 3% acetic acid test and 5% Lugol’s iodine staining. Multi-point biopsy was performed in areas exhibiting abnormal colposcopic findings, while random biopsy was conducted in regions without abnormalities. Additionally, cervical canal scratching was executed in the triple transformation area. Biopsy specimens were forwarded to the pathology department for examination, with diagnoses classified according to The Lower Anogenital Squamous Terminology (LAST) 2012 Edition (24).

#### 2.2.2 Data labeling

Labeling adhered to the standardized colposcopic terminology outlined by the International Federation for Cervical Pathology and Colposcopy (IFCPC) in 2011 and the American Society for Colposcopy and Cervical Pathology (ASCCP) in 2018 (10, 11). Lesions were categorized into low-grade squamous intraepithelial lesion (LSIL), high-grade squamous intraepithelial lesion (HSIL), and suspicious invasive carcinoma signs. Typical acetate images were selected for labeling, correlating lesion area and grade with pathological diagnostic findings.

#### 2.2.3 Annotation process

Annotation utilized the Pair annotation tool, accommodating various data modalities and formats, and encompassing annotation types such as segmentation, classification, target detection, and key point localization. Annotations included ellipses, polygons, rectangular boxes, key points, classification labels, and measurement items. Data encryption ensured safeguarding. Initial annotation was conducted by a specialist with over 5 years of colposcopy experience, followed by review from a specialist with over 10 years of experience. Consensus on lesion areas and criteria for label identification was reached between reviewers.

#### 2.2.4 Post-labeling

After excluding unclassifiable and invalid images, colposcopic images were categorized into low-grade, high-grade, and invasive cancer signs. A total of 1837 abnormal colposcopic images were obtained from the two hospitals, comprising 1124 LSIL images, 575 HSIL images, and 138 cancer images. Concurrently, 1070 normal colposcopic images were utilized to assess correct lesion classification rates. Among these, 1370 abnormal colposcopic images (838 LSIL, 428 HSIL, 104 cancer) and 800 normal colposcopic images originated from Ningbo Medical Center Lihuili Hospital. Additionally, 467 abnormal colposcopic images (286 LSIL, 147 HSIL, 34 cancer) and 270 normal colposcopic images were obtained from The First Affiliated Hospital of Ningbo University.

### 2.3 Lesion detection method of fusion image segmentation and classification

#### 2.3.1 Modeling tasks

In this research, images are labeled as Low-Grade Squamous Intraepithelial Lesions (LSIL), High-Grade Squamous Intraepithelial Lesions (HSIL), and cervical cancer uniformly as anomalous to differentiate them from normal imagery. Experiments are conducted by using Convolutional Neural Networks (CNNs) to train on these colposcopic cervical images, focusing on localizing and segmenting lesions, yielding promising findings.

#### 2.3.2 Related work

Image analysis is essential in computer vision, utilized for processing 2D images, videos, and medical data. Key to this field is image segmentation, where pixels are classified to delineate targets. Deep Learning has overtaken traditional approaches in image analysis (25). Now, Artificial Intelligence(AI) advances aid in analyzing colposcopic images for cervical cancer screening, improving diagnosis with specialized algorithms. Current research on colposcopic images encompasses various aspects, including cervical region recognition and segmentation (26, 27), image registration pre- and post-application of acetic acid (28) for detecting regions of interest (ROIs), and lesion classification (20, 29). Such studies significantly aid clinicians in colposcopy diagnoses. Deep Learning (DL) excels in extracting features through data-driven approaches. From preliminary analysis, normal images typically do not show lesion characteristics. Low-Grade Squamous Intraepithelial Lesion (LSIL) features are hard to detect due to their subtlety, whereas High-Grade Squamous Intraepithelial Lesion (HSIL) features are clearer, showcasing distinct epithelial and vascular traits. Features of carcinoma are the most pronounced among the groups, even though data for carcinoma is less abundant. There’s a clear increase in lesion severity from LSIL to carcinoma. In clinical screenings, accurately distinguishing between normal and lesional colposcopic images is essential.

#### 2.3.3 Model design

The modeling task in the proposed approach is defined as a joint image classification and semantic segmentation task within computer vision (30). The proposed feature mining approach consists of three key components: feature extraction of cervical regions, segmentation and classification of cervical lesion networks, and visualization of results. Initially, an enhanced Atrous Spatial Pyramid Pooling (ASPP) (31) method is employed to extract features of the cervical areas from images, incorporating an attention mechanism for multi-scale information fusion. Subsequently, the extracted features are fed into a decision network. Finally, the images are reconstructed and scaled to their original proportions for visual presentation of the findings. The proposed deep-decision network (DepDec) adopts a two-stage design to overcome challenges posed by limited samples in deep learning, thereby achieving superior results within a constrained dataset.

#### 2.3.4 Overview of the architecture

**Figure 1:**
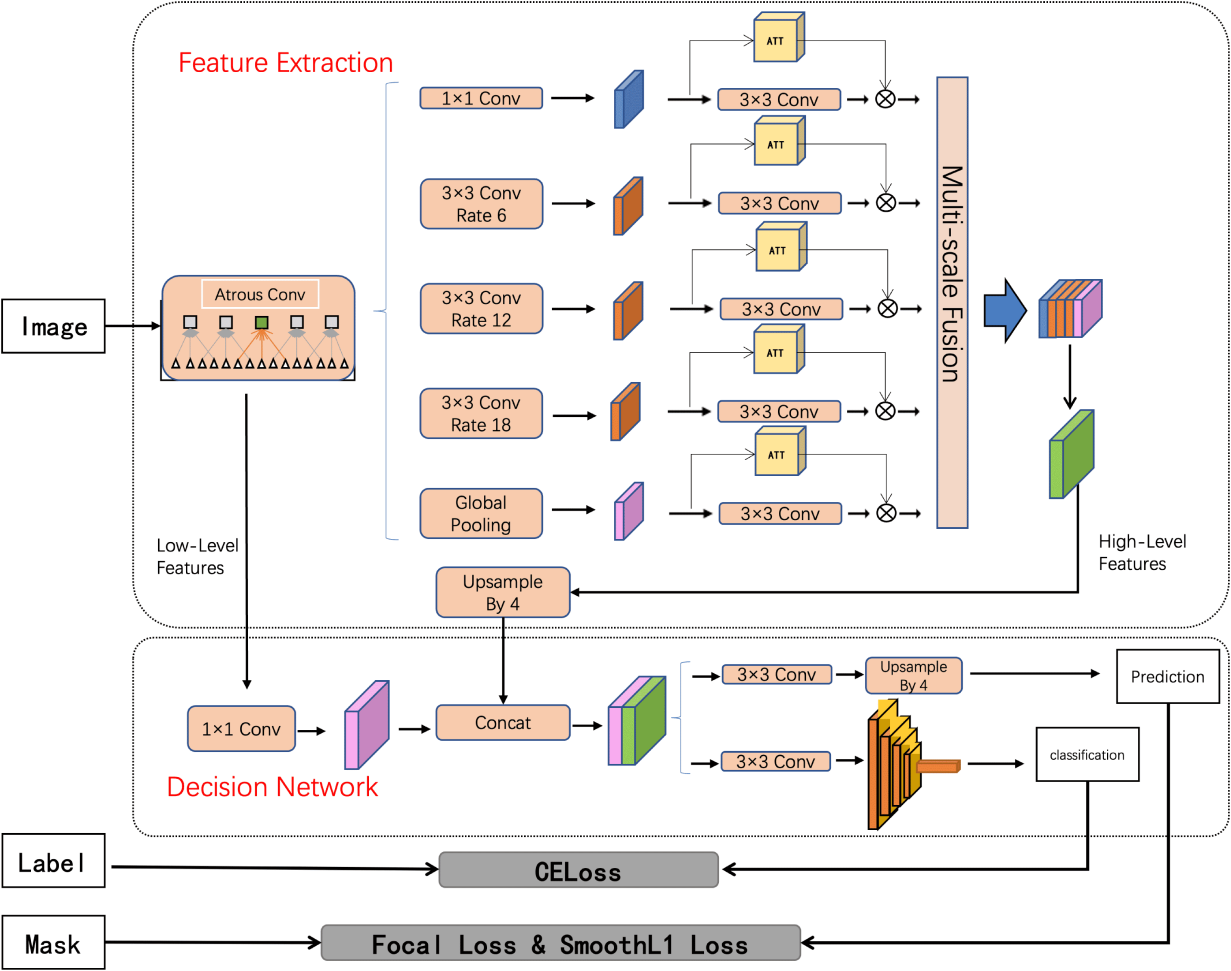
Schematic diagram of DepDec model network structure.

The comprehensive architecture of the DepDec model is depicted in **Figure 1**. Structured around two core modules, namely Feature Extraction and Decision Network, DepDec presents a robust feature mining framework for image analysis.

#### 2.3.5 Feature extraction

The proposed approach employs the Deep Residual Network with 101 layers (ResNet101) network as the backbone for extracting cervical lesion features. Incorporating an attention mechanism at multiple scales, this approach enhances the discriminative capability of the model, allowing it to focus on relevant features and suppress the less informative ones, thereby improving the accuracy of lesion detection and classification.

Specifically, as shown in **Figure 1**, layer1 of ResNet101 is employed as the low-level features, and layer4 is used as the feature map input for Atrous Spatial Pyramid Pooling (ASPP) (31). The formula is as follows:


(1) $$y\left[i\right]=\sum_{k=1}^{K}x\left[i+r\cdot k\right]w\left[k\right].$$

Atrous convolution as a powerful tool, allows the work to explicitly control the resolution of features computed by deep convolutional neural networks and adjust the receptive field of filters to capture multi-scale information. It extends the capabilities of standard convolutional operations. To further enhance the representational power of the network, this study introduce an attention mechanism (32) after each convolutional layer. The attention mechanism aims to selectively emphasize informative features and suppress less relevant ones, enabling the network to focus on important regions of the input. Subsequently, the low-level features are dimensionally adjusted using 1×1 convolutions, and the high-level features are upsampled four times. Finally, the low-level and high-level features are fused to incorporate semantic information.

The network employs techniques such as enlarging the receptive field, utilizing contextual information, and integrating multi-scale features to obtain deep semantic information, thereby establishing the foundation for achieving pixel-level localization and predictive segmentation of vaginoscopic images.

#### 2.3.6 Decision network

It contains two key components. The first component is the prediction head, which performs segmentation by utilizing 3× 3 convolutions and bilinear interpolation to accurately identify the colposcopic lesion region.

The second component is a discriminator, comprising a deep residual network with internal residual blocks (33). The formula is as follows:


(2) $$O_{h}^{i}=a_{k}^{i}I_{h}^{i}+b_{k}^{i},\forall_{i}\in w_{k}$$

that incorporate jump links. This design effectively addresses the challenges associated with neural network depth and significantly enhances accuracy. The discriminator is responsible for discriminating whether the entire image exhibits anomalies.

#### 2.3.7 Two-stage approach

The study employs a decision network comprising a segment network and a discriminator. The segment network deciphers deep features through convolution, pooling, and upsampling layers, enabling precise pixel-level segmentation. The formula is as follows:


(3) $$O_{h}={\bar{a}}_{i}\times I_{h}^{i}+{\bar{b}}_{i}$$

For lesion localization, it condenses the image’s characteristics into a one-dimensional vector. Experimental findings underscore the pivotal role of high-level semantic information in classification.

To tackle overfitting, the study conducts the two stages independently. Initially, the pixel segmentation network undergoes exclusive training. Subsequently, the segmentation network’s weights are fixed, and the focus shifts to fine-tuning the discriminator. This approach ensures effective learning of the discriminator while minimizing the risk of overfitting associated with the segmentation network’s extensive weight parameters.

### 2.4 Loss function

In the experimental procedure, this study employ both the focal loss and the smooth L1 loss to compute the segmentation loss. Additionally, the study utilize the cross-entropy loss function to compute the classification loss. The designed loss function is as follows:


(4) $$\begin{array}{c} Loss\;=-\frac{1}{N}\sum_{i=1}^{N}(\lambda_{{ℒ}_{1}}\text{Smooth}(y_{0}^{\left(i\right)},\hat{y_{0}^{\left(i\right)}})\\ \;\;\;+\lambda_{{ℒ}_{\text{focal}}}\text{Focal}(y_{0}^{\left(i\right)},\hat{y_{0}^{\left(i\right)}})+y_{1}^{\left(i\right)}\log\left(\hat{y_{1}^{\left(i\right)}}\right)) \end{array}$$

Where *N* represents the total number of samples. $y_{0}^{\left(i\right)}$ denotes the pixel predictions for the entire image, while $\hat{\;y_{0}^{\left(i\right)}}$ represents the corresponding ground truth, $\lambda_{{ℒ}_{1}}$ and $\lambda_{{ℒ}_{\text{focal}}}$ are the balancing hyper-parameters. $y_{1}^{\left(i\right)}$ represents the classification prediction, and $\hat{y_{1}^{\left(i\right)}}$ represents the corresponding ground truth, where $y_{*}^{\left(i\right)}$ is the *i*-th input image.

### 2.5 Experimental procedures

The experimental framework was executed using PyTorch 1.9.1 within a Compute Unified Device Architecture (CUDA) 11.1 environment on a single 3060 GPU. To circumvent the constraints imposed by the graphics processing unit (GPU) memory limitations, the study divided the batch size into four sample layers. However, each pixel segmentation layer of the image was treated as a separate training entity, effectively augmenting the effective batch size.

This study performed fundamental image preprocessing techniques, such as resizing, rotating, and flipping, to prepare the data. Additionally, data augmentations including rotation and shearing (34) are employed to enhance the diversity of the experimental dataset and tackle issues related to data imbalance among samples, thus simulating real-world scenarios. The original lesion annotations were retained and converted into binary masks to facilitate the semantic segmentation task of the images.

To assess the model’s performance and mitigate the risk of overfitting, this study implemented five-fold cross-validation. The dataset was split into an 80% training set and a 20% validation set. We apply cross-validation and combine the test results from each fold to present a comprehensive outcome, which reflects the aggregated performance across all test sets after five experimental runs. Leveraging transfer learning, the study leveraged the ResNet101 pre-trained model from the ImageNet dataset as the feature extractor.

For the detection of lesion regions (LSIL, HSIL, cancer), the study fine-tuned the network using an attention mechanism embedded within the semantic segmentation framework. This attention mechanism bolstered the representation capability of the feature pyramid, consequently enhancing the depiction of deep semantic features.

Experiments were conducted utilizing the DeepLabv3+ model as the foundation for the initial semantic segmentation of image data. The attention mechanism was subsequently incorporated for fine-tuning to enhance the generalization capacity of the segmentation network. The target output of the segmentation network was identified as the lesion region, with the following parameters: Batch=4, Epoch=200, ResNet101 backbone network parameters training.

The parameters acquired from training were utilized to predict the lesion region on the validation set of colposcopy images. Subsequently, the prediction result map generated by the algorithm was juxtaposed with the lesion region labeled by medical professionals. A confusion matrix was then constructed to derive the mean intersection over union (mIOU), mean average precision (mAP), true positive rate (TPR), false positive rate (FPR), and receiver operating characteristic curve (ROC curve) at various thresholds.

Furthermore, this study deliberated on the two-stage learning mechanism for the segmentation network and the decision network, along with the design of the corresponding loss function. During the experiment, data were randomly sampled for training purposes. Ultimately, the average cross-merge ratio mIOU was determined to be 0.6051.

## 3 Results

This study primarily focused on the dichotomous classification and localization of lesions (LSIL, HSIL, and cancerous lesions) alongside normal tissue, aiming to achieve accurate classification aligning with pathological judgment. Notably, this experimental model exhibited a high level of agreement with pathological assessments in classifying lesions.

To mitigate the potential bias stemming from a single center, the study adopted two distinct datasets for experimentation and testing, thereby bolstering the generalizability and reliability of the findings. The incorporation of multiple datasets facilitated robust result analysis and bolstered the credibility of the conclusions.

This study employed a five-fold cross-validation approach to derive average results. The training and validation sets were randomly selected five times, maintaining an 8:2 ratio for dichotomous classification. An artificial intelligence model was trained to differentiate between normal and lesion images. Subsequently, the detection outcomes of the test set images were juxtaposed with expert labeling, and the average of the five results was computed. Performance assessment was conducted through ROC curves plotted at various thresholds, with the true positive rate (TPR) on the vertical axis and the false positive rate (FPR) on the horizontal axis. The area under the ROC curve (AUC) served as a performance metric to evaluate the model’s efficacy.

**Figure 2:**
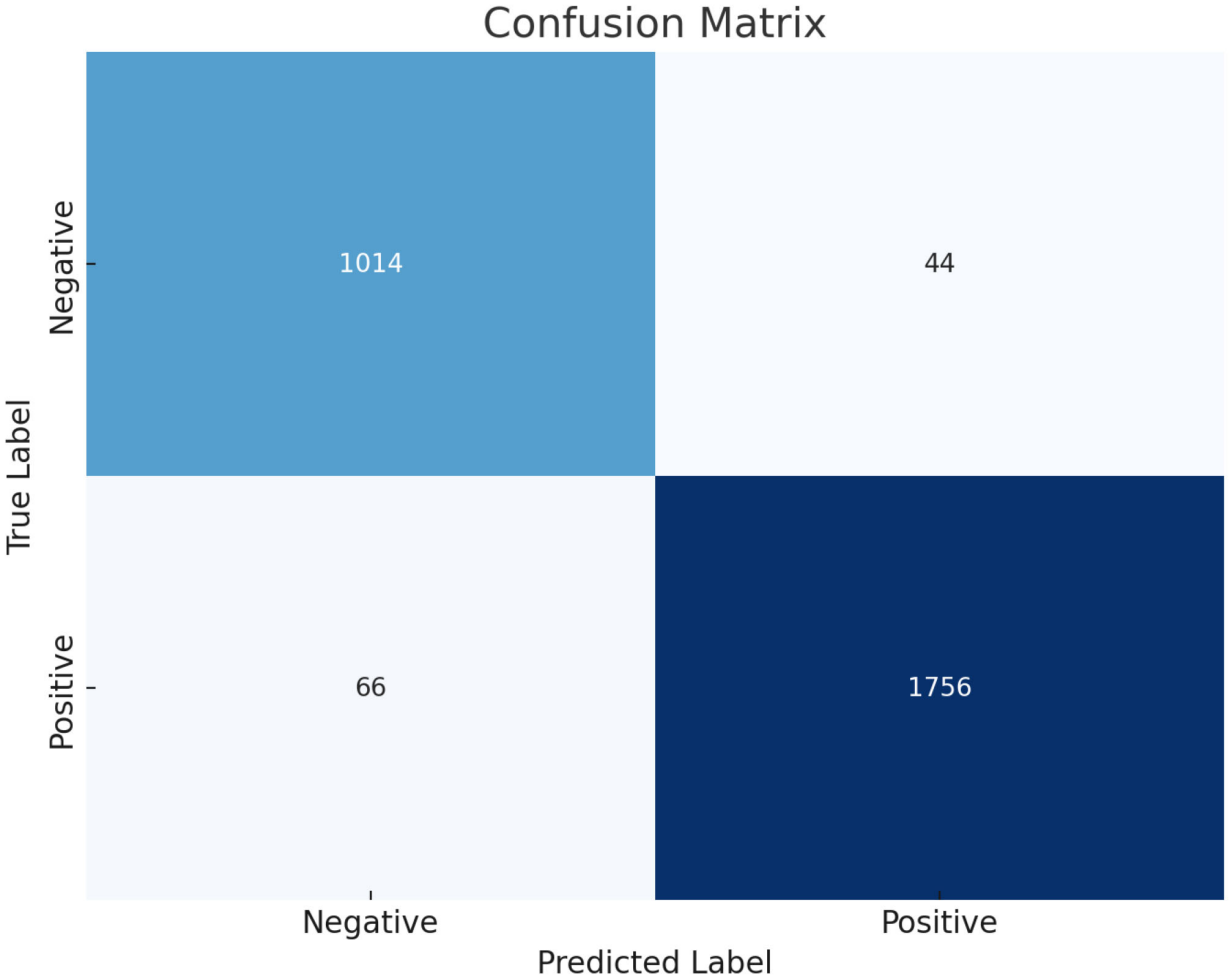
Confusion matrix on the multi-center data set with cross validation.

The results, as depicted in **Figure 2**, elucidate the fluctuation of the curve when the training set is partitioned into different subsets. This sheds light on how variations in the training data influence the classification output and the variance observed between the splits generated by cross-validation. Furthermore, leveraging confusion matrices enabled a deeper analysis of this model’s efficacy. Considering lesions as positive instances, the study quantified the true negatives (TN), false positives (FP), false negatives (FN), and true positives (TP) as 1014, 44, 66, and 1756, respectively. These findings underscore the model’s commendable ability to discriminate between normal and lesion cases.

**Figure 3:**
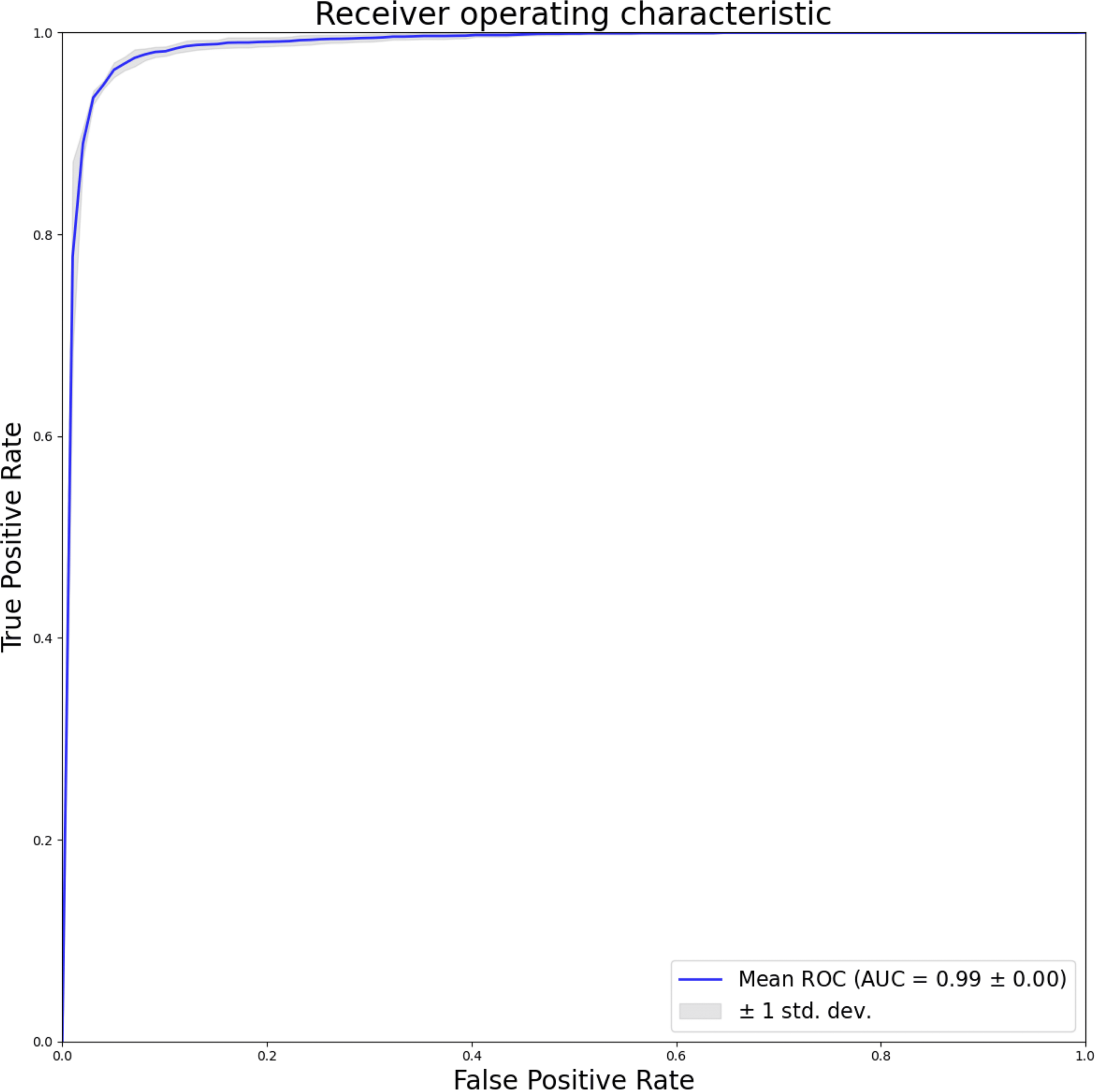
Image level dichotomous ROC curves to distinguish normal from lesions.

This study presents a comprehensive evaluation of the model’s efficacy in discriminating between normal and lesion images. The model demonstrates an impressive accuracy of 96.18%, indicating notable levels of sensitivity, specificity, precision, and F1 score, measuring at 96.38%, 95.84%, 97.56%, and 96.96%, respectively. Additionally, the study conduct a thorough assessment of the model’s performance using image-level ROC curves, illustrated in **Figure 3**. The considerable area under the ROC curve yields an AUC of 0.99, underscoring the model’s capacity to distinguish between normal and lesion cases while exhibiting resilience to variations in the training data. These findings affirm the model’s feasibility and its potential to inform clinical decision-making and validation processes.

**Figure 4:**
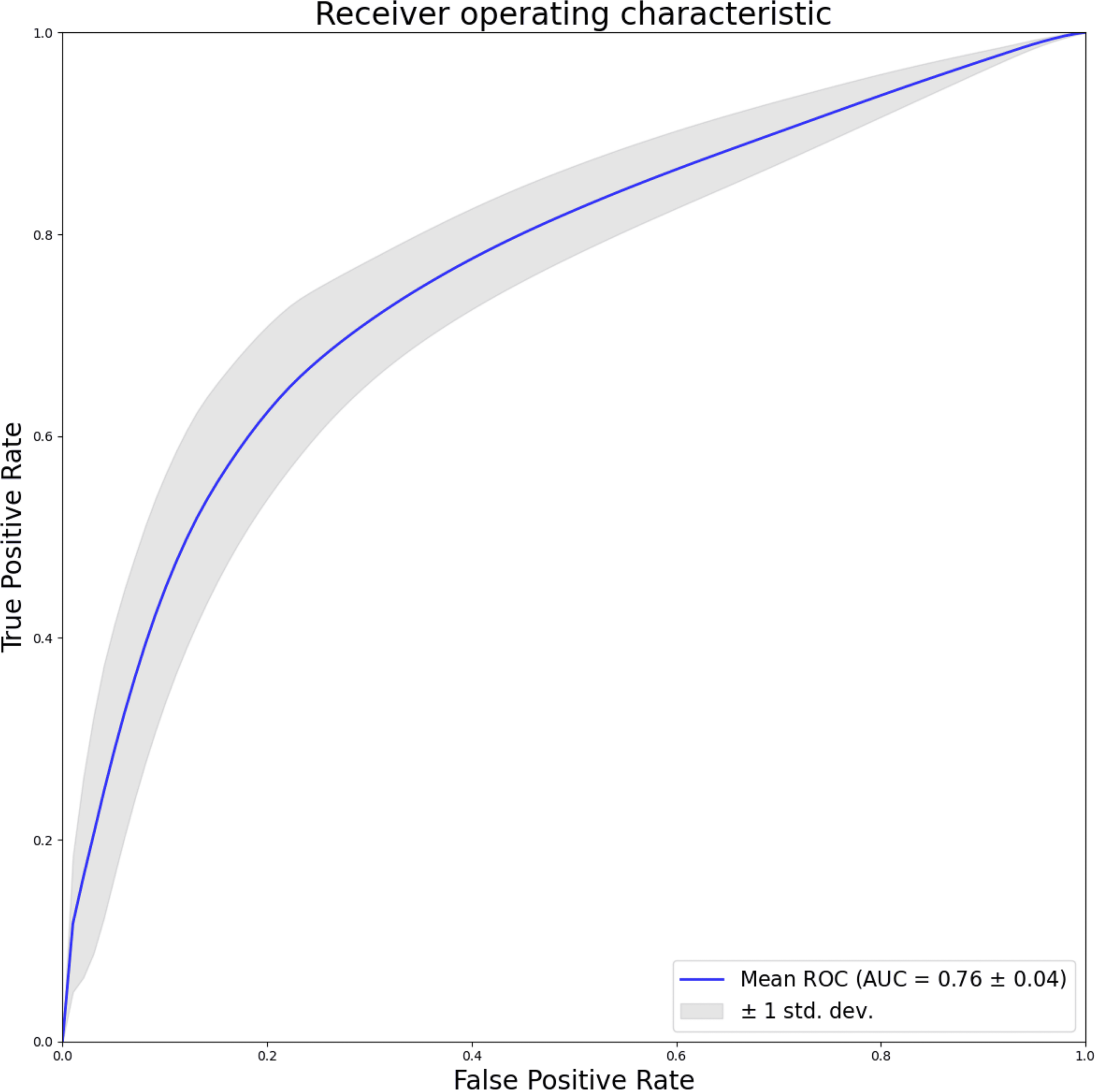
Pixel level dichotomous ROC curves to distinguish normal from lesions.

Furthermore, this study utilize pixel-level ROC curves and actual predictions, depicted in **Figure 4**, to evaluate the model’s localization capabilities. The resulting AUC of 0.76, with a standard deviation of 0.04, signifies the model’s efficacy on a smaller-scale dataset in accurately localizing lesions. This aspect of the evaluation provides valuable insights into the model’s performance at a granular level, contributing to a more comprehensive understanding of its capabilities.

In accordance with recent literature outlined in **Table 1**, deep learning methodologies have seen widespread application in the recognition of colposcopic normal and lesion images. This model exhibits an impressive accuracy of 96.18% in the multi-center setting, effectively discerning between normal and lesion images, with associated sensitivity, specificity, precision, and F1 score metrics of 96.38%, 95.84%, 97.56%, and 96.96%, respectively.

**Table 1:** Summary of literature on colposcopic lesion recognition based on AI methods.

Method	Year	Data set	Research objectives	Accuracy rate _%_
Asiedu (37) Support vector machines	2018	Acetic acid and iodine images	CIN (Cervical Intraepithelial Neoplasia) and non-CIN	80.0%
Yuan, Chun-Nan (38) Multimodal Resnet Model	2020	10365 normal, 6357 LSIL, 5608 HSIL cases.(Each data item includes One saline image, one cetate image,one iodine image)	Normal and lesions	84.10%
Yinuo Fan (39) CMF-CNN	2022	3093 normal,2794 LSIL, and 1219 HSIL + cases.	Normal and lesions	92.70%
Zhen Li (40) Deeplabv3+	2023	339 high-level cervical lesions and 313 microinvasive or invasive cervical cancer.	HSIL and Cancer	93.29%
Yung-Taek Ouh (41) CerviCARE AI	2024	11,500 Negative images and 11,225 Positive images	Negative and Positive	84.3%
Ours DepDec model	–	1070 normal and 1837 lesioned case acetate images	Normal and lesions	96.18%

As illustrated in **Table 2**, to assess the performance of the proposed approach in localizing cervical lesion images, the study conducted a comparative analysis involving the U-net++, DeepLabv3+, and DepDec models, utilizing a dataset comprising colposcopic acetic acid images of lesion cases. Through extensive experimentation, the study opted to enhance the DeepLabv3+ model by augmenting the colposcopic acetic acid image data and fine-tuning the parameters. Consequently, the DepDec model achieved comparable accuracy in lesion area segmentation, yielding a segmentation accuracy (mAP) of 91.85% with a reduced sample size. This performance surpassed that of both the U-net++ and DeepLabv3+ models.

**Table 2:** Comparison of lesion area localization based on DL colposcopic acetate images.

Method	Data set	Research objective	Segmentation accuracy
U-net++	1837 acetic acid images of lesion cases	Lesion area localization	86.00%
DeepLabv3+	1837 acetic acid images of lesion cases	Lesion area localization	86.76%
DepDec model	1837 acetic acid images of lesion cases	Lesion area localization	91.85%

**Figure 5:**
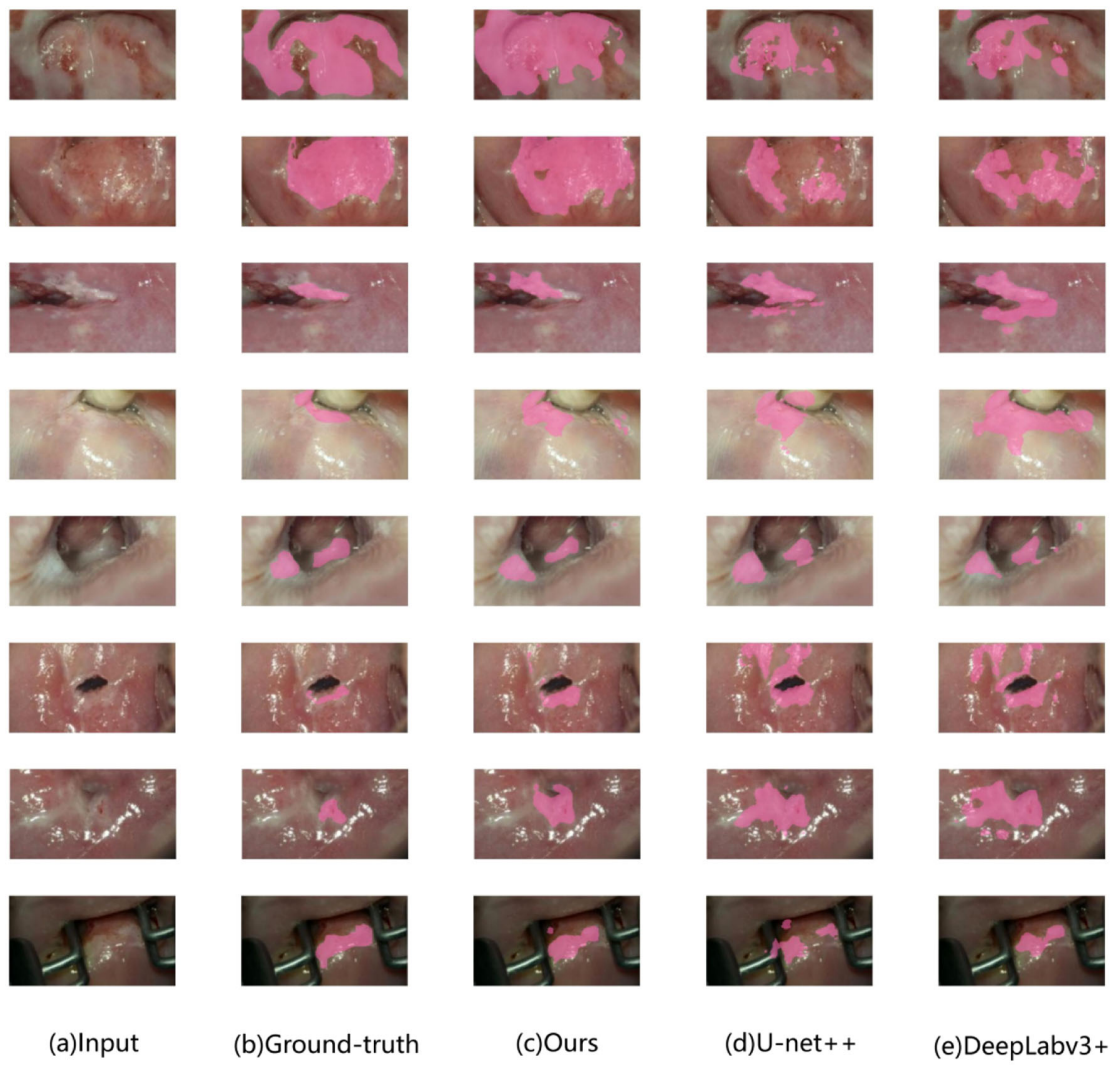
Example of model output comparison on the validation set. Lesion image: pink masked area indicates lesion area. **(A)**: colposcopic acetate image. **(B)**: physician-labeled lesion area. **(C)**: lesion areas identified by the proposed approach. **(D)**: those by U-net++. **(E)**: those by DeepLabv3+ respectively.

Illustrated in **Figure 5** are some examples of detection results by different approaches. From left to right, the images depict the vaginoscopy image from the validation set, the physician’s annotated region serving as the ground truth label, and the predicted region generated by deep learning models. Notably, results from the proposed approach are more close to the ground truth, and thus, generally performs better than others.

## 4 Discussion and conclusion

Cervical cancer screening, as advocated by the World Health Organization, primarily involves HPV screening, cytology, and colposcopy, with combined cytology and HPV screening emerging as the preferred method presently (35). However, histological examination through colposcopic cervical biopsy remains the gold standard for diagnosing cervical disease. The accuracy of colposcopic diagnosis significantly hinges on the operator’s experience, highlighting the importance of enhancing colposcopy and biopsy accuracy in managing cervical lesions (36).

Given the significance of timely diagnosis and treatment amidst the high prevalence of cervical cancer, scholars are actively engaged in devising accurate and cost-effective screening and diagnostic techniques. With the evolution of AI technology, its integration into medical diagnosis has witnessed considerable advancement, particularly in cervical lesion detection. This study endeavors to construct a model capable of not only identifying lesions but also localizing and annotating lesion regions to guide clinical colposcopy, biopsy, and localization treatment.

Utilizing a diverse range of cervical epithelial and vascular signatures in internationally standardized colposcopy terminology, this study semantically annotates images at the pixel level. Leveraging a pre-trained network based on transfer learning as a feature extractor, this study develop a multi-scale deep decoding feature-based decision network model with a two-stage architecture incorporating image segmentation and classification methods. Through interdisciplinary collaboration, the study synthetically compare and optimize DL network architectures of multiple classical semantic segmentation models, focusing on aspects such as data preprocessing, enhancement, backbone network selection, training, hyperparameter optimization, and algorithm enhancement to outperform traditional single classification or segmentation models.

The primary objective of this model is to effectively differentiate between normal and pathological cases. Normal cases, ascertainable by physicians without biopsy, warrant close follow-up and are suitable for clinical screening. The model facilitates clinical triage, reducing unnecessary patient biopsies and averting excessive medical interventions. In instances of diseased cases, upon identifying pathological instances and automatically delineating suspicious diseased areas, this model aids physicians in precisely locating recommended diseased areas and determining lesion grades through biopsy combined with their clinical judgment. Furthermore, it provides guidance for subsequent localization treatments.

Comparison between **Tables 1** and **2** underscores the commendable performance of the research model in colposcopic image classification and lesion localization, surpassing comparison models in accuracy and segmentation accuracy.

This study presents several notable contributions. Firstly, in terms of experimental validation, the model integrates deep decoding features and residual information, resulting in a pixel-based model with enhanced discriminative power for distinguishing between normal and abnormal images compared to others. Secondly, the model effectively captures rich contextual information by employing pooling features at various resolutions to delineate clear objects, while utilizing deep decoding features to augment classification accuracy through the addition of considerable depth via residual layers. In a multi-center setting, this model achieves an accuracy of 96.18%, with notable sensitivity (96.38%), specificity (95.84%), precision (97.56%), and F1 score (96.96%). These findings demonstrate the model’s capability to discern differences between normal and lesioned cases, aligning closely with pathological judgment. Additionally, the model accurately estimates and delineates lesion areas, thereby aiding physicians in classification judgment and guiding biopsy and subsequent localization treatment. Notably, the model’s performance surpasses that of experienced colposcopy specialists trained in IFCPC terminology, suggesting its potential to enhance diagnostic accuracy and guide treatment decisions.

This study, while yielding relatively high classification accuracy through the use of colposcopic images from different instruments across two hospitals to mitigate bias, reveals certain limitations. Firstly, the generalizability of the findings may be constrained despite efforts to diversify sources. Future endeavors could enhance model performance through the inclusion of samples from a wider array of centers, thereby broadening sample diversity and size, as well as refining model training. Secondly, the current model does not differentiate among lesion severities such as LSIL, HSIL, and cancerous lesions. Prospective studies could benefit from integrating multiple clinical indicators, including TCT/HPV data, and employing multimodal features for a more precise prediction. Additionally, by engaging physicians in annotating lesions with finer segmentation categories, more nuanced segmentation and interpretations can be achieved based on this model, offering improved diagnostic guidance. Developing techniques for better handling intra-class variability could improve model accuracy and clinical applicability.

In summary, this model effectively distinguishes between normal and lesioned cases, aiding in lesion localization and guiding treatment decisions. Future efforts will focus on refining classification training and developing a comprehensive AI-aided diagnosis system for colposcopy, which holds promise for improving diagnostic accuracy and guiding treatment strategies, particularly for inexperienced practitioners.

## Data Availability

The raw data supporting the conclusions of this article will be made available by the authors, without undue reservation.
